# Metamaterial Lensing Devices

**DOI:** 10.3390/molecules24132460

**Published:** 2019-07-04

**Authors:** Jiangtao Lv, Ming Zhou, Qiongchan Gu, Xiaoxiao Jiang, Yu Ying, Guangyuan Si

**Affiliations:** 1 College of Information Science and Engineering, Northeastern University, Shenyang 110004, China; 2 College of Information & Control Engineering, Shenyang Jianzhu University, Shenyang 110168, China; 3 Melbourne Centre for Nanofabrication, Clayton, Victoria 3168, Australia

**Keywords:** metamaterial, nanofocusing, perfect lens, metasurfaces

## Abstract

In recent years, the development of metamaterials and metasurfaces has drawn great attention, enabling many important practical applications. Focusing and lensing components are of extreme importance because of their significant potential practical applications in biological imaging, display, and nanolithography fabrication. Metafocusing devices using ultrathin structures (also known as metasurfaces) with superlensing performance are key building blocks for developing integrated optical components with ultrasmall dimensions. In this article, we review the metamaterial superlensing devices working in transmission mode from the perfect lens to two-dimensional metasurfaces and present their working principles. Then we summarize important practical applications of metasurfaces, such as plasmonic lithography, holography, and imaging. Different typical designs and their focusing performance are also discussed in detail.

## 1. Introduction

In recent years, surface plasmons and related devices [1,2,3,4,5,6,7,8,9,10,11,12,13,14,15,16,17,18,19,20,21,22,23] have been thoroughly investigated due to their potentially wide applications in nanophotonics [24,25,26,27,28,29,30,31,32,33,34,35,36,37,38], biology [39,40,41,42,43,44,45], spectroscopy [46,47,48,49,50,51], and so on. They are capable of manipulating electromagnetic waves [52,53,54,55,56,57,58,59,60,61,62] at the nanometer scale to achieve all-optical integration, providing an effective way to develop smaller, faster and more efficient devices. Surface plasmon resonance is based on the oscillation of electrons formed at the metal-dielectric interface. The resonant wavelength can be continuously tuned from ultraviolet to infrared, enabling various practical devices [63,64,65]. These devices have gained wide attention and experienced considerable development because they have small dimensions, fast operating speed, and low energy consumption. In particular, lensing and focusing components are widely used in many technologies, such as high-resolution imaging [66,67,68,69,70], nanolithography [71,72,73,74,75], and optical integration [76,77,78,79]. However, due to the diffraction limit which means the resolvable feature size is determined by$\;d=\frac{\lambda }{2\mathrm{NA}}$ (where *d* is the resolvable feature size, *λ* is the wavelength of incident light, and NA is the numerical aperture) due to the wave nature of radiation, the imaging resolution of conventional lenses and optical systems is difficult to break through the dimension of half of the incident wavelength. Therefore, it is of great scientific and application significance to break through the diffraction limit and achieve super-resolution focusing and imaging effects. Some information of the subwavelength structure (the object) is hidden in the evanescent wave. The intensity decays rapidly with increasing distance from the object and the attenuation speed is proportional to the spatial frequency. Recently, more metamaterial-enabled technologies have been reported, including computing metastructures [80], near-zero-index wires [81], curvilinear metasurfaces for surface wave manipulation [82], acoustic resonators [83], and so on [84,85]. 

Although Veselago first proposed a negative index medium in 1968 [86], a new concept of a perfect lens proposed by Pendry [87] started to draw great attention after about 30 years, which enabled the scientific community to re-understand and examine the abnormal electromagnetic properties and cloaking effects of metamaterial media. Later, Fang et. al. experimentally demonstrated the superlensing effect using an evanescent wave through a silver film [88]. Then the same group investigated the far-field superlensing effect [89] and proposed a hyperlens design. Conventional optical lenses focus by adjusting the phase of the incident light by varying the thickness of optical materials (e.g., glass). Such lenses are bulky and cumbersome. With the continuous development of micro-nano optics, traditional optical lenses are difficult to meet the requirements of large-scale integration and device miniaturization and functional diversification. Metasurface lenses [90,91,92,93,94] are made of two-dimensional (2D) planar structures that artificially arrange optical antennas with special electromagnetic characteristics in a certain way, which can achieve flexible regulation of amplitude, phase, polarization and other parameters of the incident light. Important applications, such as holographic optics and achromatic lenses, have been triggered. In addition, these 2D planar structures are easier to process and integrate, providing solutions for miniaturization and integration of optical lenses. Here, we review the metamaterial superlensing devices (Section 2) and two-dimensional metasurfaces (Section 3) working in transmission mode from ultraviolet to visible range. We then summarize their important practical applications (Section 4), such as plasmonic lithography, holography, and imaging.

## 2. Superlensing Effect and Far-Field Hyperlens

### 2.1. Perfect Lens

Due to the diffraction limit, the imaging resolution of conventional optical lenses can only reach half of the wavelength of the incident light. By recovering and enhancing the high-frequency evanescent wave carrying detailed information of the object, perfect lenses based on metamaterials with negative refractive indices are expected to break through this optical diffraction limit and achieve super-resolution imaging. Due to the loss of light waves propagating in metal, how to enhance the high-frequency evanescent wave signal more efficiently and convert it into a propagating wave to participate in imaging to better achieve far-field super-resolution imaging and how to further increase the near field ultra-high resolution focused spot depth and reduce the far field focused spot size are the focus of further research on surface plasmon-enabled lenses. Pendry first proposed the perfect lens theoretically using negative index media [87], which can amplify the evanescent waves. A perfect lens with a negative refractive index (negative permittivity and permeability) can focus both propagating and evanescent waves. Different from the traditional optical lens, a superlens utilizes the negative refraction effect of the medium to not only converge the propagation wave of the far-field transmission, but also to recover and enhance the near-field evanescent wave signal.

**Figure 1:**
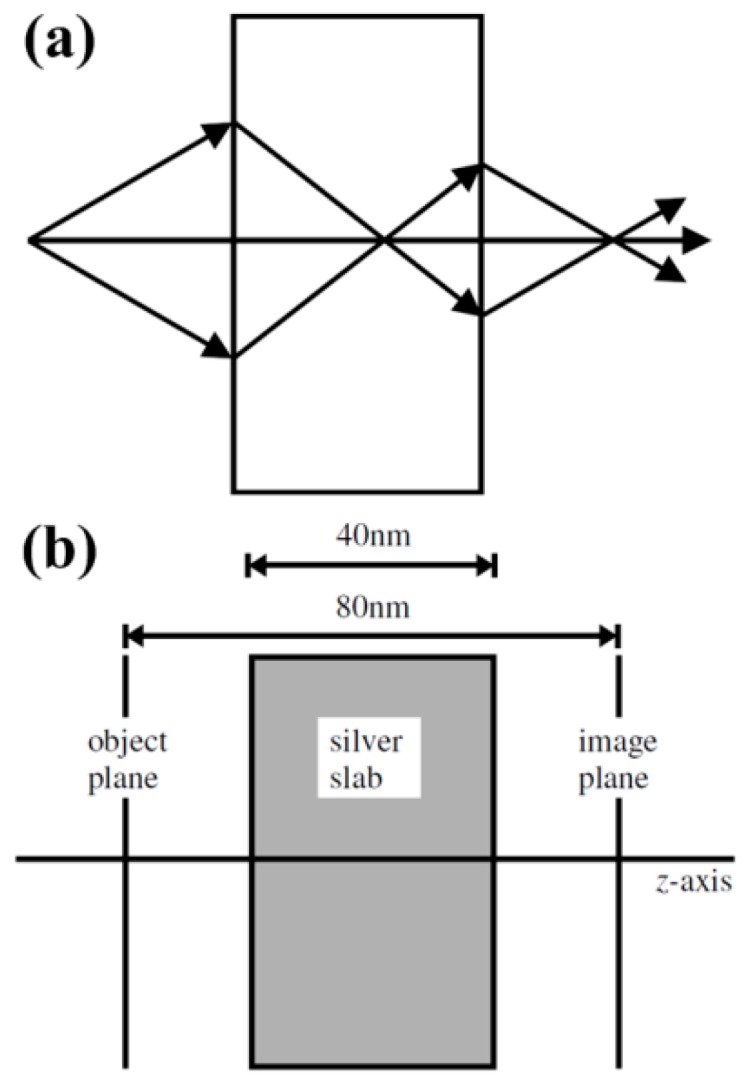
Pendry’s perfect lens. (**a**) Schematic drawing of a parallel-side slab with a negative refractive index acting as a perfect collecting lens. (**b**) A proposed silver slab lens with dimensions and object/image plane labeled. Reproduced with permission from [87]. Copyright American Physical Society, 2000.

As shown in Figure 1a, since the metamaterial (artificial medium) has a negative refractive index and an evanescent wave amplification characteristic, the evanescent wave (Fourier component) emitted by the electromagnetic wave source will not only be corrected in phase but also its amplitude will be effectively amplified. This can lead to perfect recovery of the object’s information at the image point [95,96,97], breaking the diffraction limited resolution of traditional lens, and achieving the purpose of super-resolution (sub-wavelength) focusing of electromagnetic waves. Figure 1b shows an example of a silver slab lens in operation. Although only the imaginary part of the dielectric function prevents ideal reconstruction, a significantly improved focusing effect can still be realized.

**Figure 2:**
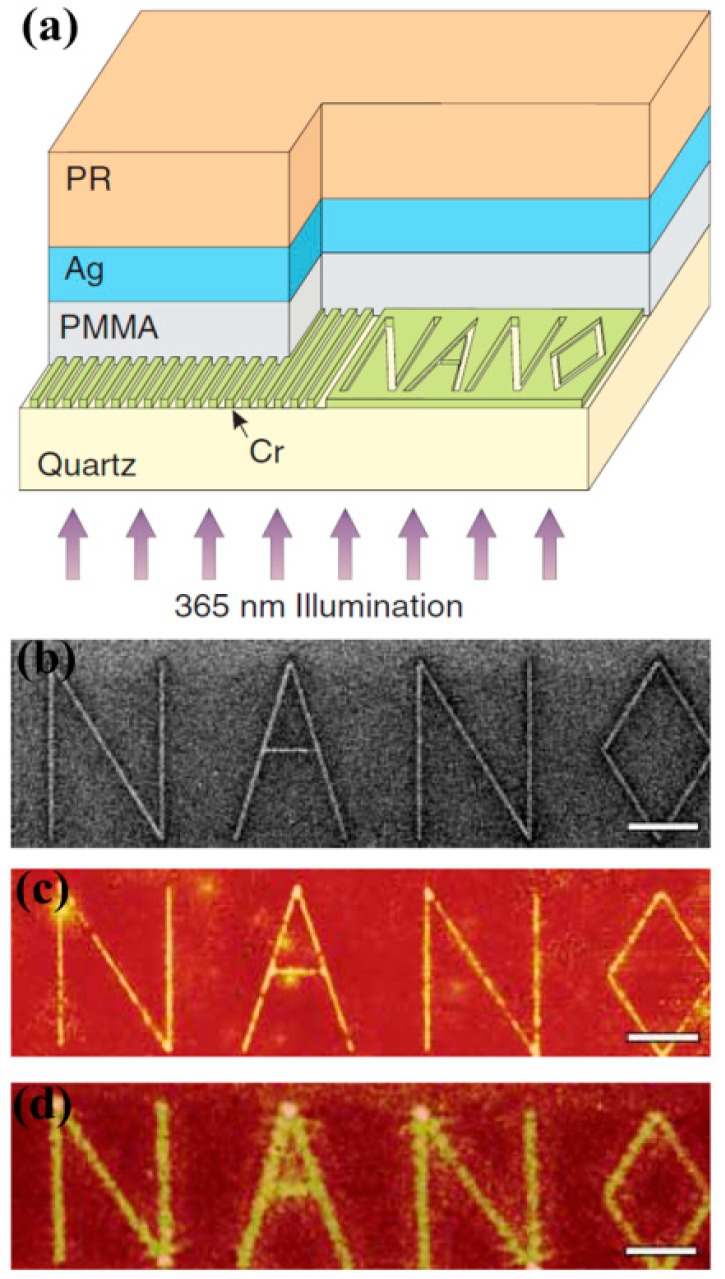
(**a**) Schematic of a silver superlens. (**b**) Object of “NANO” milled by FIB. (**c**) High resolution and fine features with the superlens. (**d**) Low resolution and blurry features without the superlens. Reproduced with permission from [88]. Copyright the American Association for the Advancement of Science, 2005.

Figure 2 shows a schematic diagram of the super-resolution imaging structure of a silver film superlens developed by Fang and coworkers [88]. Different nanostructures etched in the chromium film by using focused ion beam (FIB) are employed as the imaged objects, and the polymethyl methacrylate (PMMA) layer is used to control the spacing between the chromium film and the silver film superlens. The silver film has a thickness of 35 nm, and the image of the object is recorded on the negative photoresist layer. After development, the pattern of the object image is characterized by atomic force microscopy (AFM). Under the action of incident light with a wavelength of 365 nm, the silver film superlens amplifies the evanescent wave by exciting the surface plasmons, compensating for the exponential decay of the evanescent wave in air propagation, thus realizing super-resolution imaging. As shown in Figure 2c, under the action of the silver film superlens, the objects (“NANO” milled by FIB as shown in Figure 2b) can be clearly identified and the image is sharp and fine. However, when the PMMA layer is used in place of the silver film, the imaging result loses the fine information of the slit array etched on the chromium film, which is blurry as shown in Figure 2d. The silver film superlens achieves a fine imaging width of 89 nm (λ/4.1), and without the silver film superlens, the imaging width is (321 ± 10) nm, which is much larger than the imaging size of the former. 

### 2.2. Hyperlens with a Far-Field Superlensing Effect

**Figure 3:**
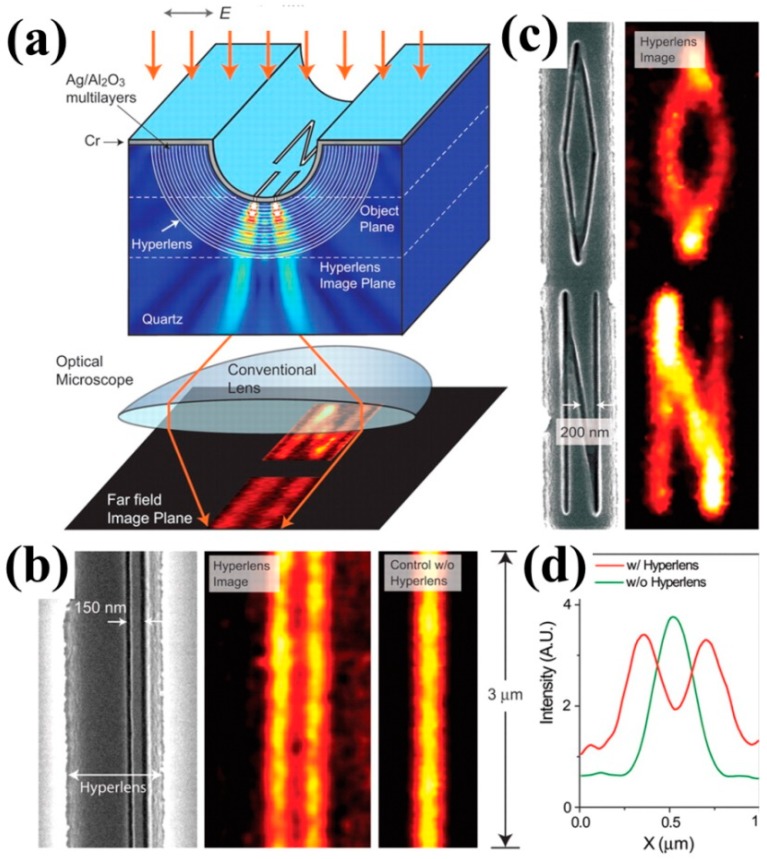
(**a**) Schematic of the multilayer hyperlens and its working mechanism. (**b**) SEM image of the slits and far-field images with and without hyperlens. (**c**) SEM image of letters “O” and “N” and far-field image with hyperlens. (**d**) Averaged cross-section of both hyperlens image and the control case without the hyperlens. Reproduced with permission from [89]. Copyright the American Association for the Advancement of Science, 2007.

After achieving the near-field superlensing effect, how to further project the near-field image into the far-field becomes critical. A hyperlens (an anisotropic metamaterial with a hyperbolic dispersion) can realize far-field super-resolution imaging with low losses, which has shown notable potential for real-time biomolecular imaging and nanolithography. Although the silver film can dramatically improve the imaging quality of nanostructures and achieve super-resolution imaging, its working distance is very small and can only be limited to the near field region since the evanescent wave rapidly decays away from the surface of the silver film superlens. Liu et al. further proposed and experimentally verified a hyperlens design that can achieve far-field super-resolution imaging effect [89]. The near-field superlens consists of a single layer of ultra-thin silver film, while the far-field hyperlens adds a periodic grating structure to the ultra-thin silver film, as shown in Figure 3a. The ultra-thin silver film enhances the evanescent wave carrying the fine structure information of the object by exciting the surface plasmon, and then converts the enhanced evanescent wave into a propagating wave by using a periodic grating structure. Figure 3b shows the experimental results of a pair of nanoslits with a width of 35 nm and 150 nm spacing and their far-field imaging. Conventional optical microscopes without the hyperlens cannot clearly capture the image of the pair of nanoslits. However, when a far-field superlens is used for imaging, the set of nanoslits can be clearly distinguished (Figure 3b hyperlens image). It can be seen that surface plasmons play a decisive role in super-resolution imaging of superlenses. Figure 3c shows arbitrary objects (letters of “O” and “N”) with ~40 nm line widths and, again, one can see that sub-diffraction resolution can be obtained. Figure 3d plots the averaged cross-section of both hyperlens image and the control case without the hyperlens. 

### 2.3. Planar Lensing with Plasmonic Gratings

**Figure 4:**
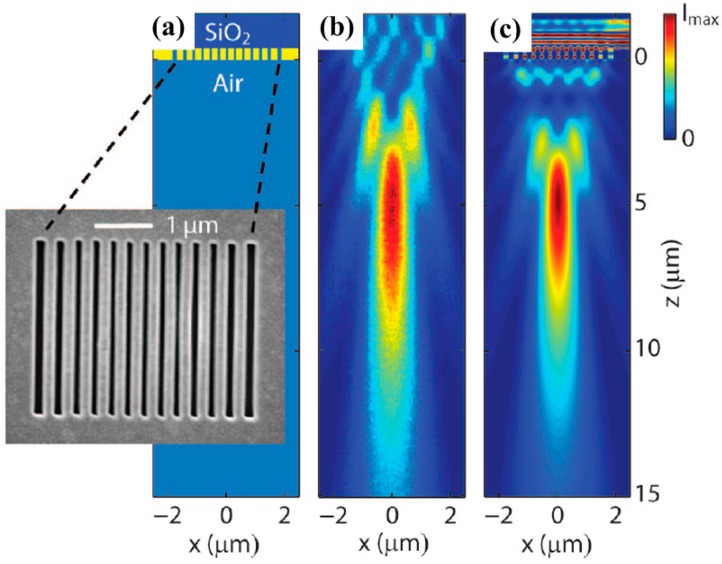
(**a**) Schematic and SEM image of the nanoslit array plasmonic lens. (**b**) Experimental results of transmitted intensity distribution captured by a confocal microscope. (**c**) FDTD-calculated result showing the intensity distribution which agrees well with measurements. Reproduced with permission from [103]. Copyright American Chemical Society, 2009.

The main limitation for current superlens and hyperlens designs is that most of them need very complex designs and some of them have very limited functional wavelength range. Simple designs with dynamic engineering functionalities are desirable. To simplify lens designs with acceptable cost and test their working principles, different analytical models have been used to verify the superlensing mechanism, such as finite difference time domain (FDTD) method and finite element method (FEM). However, these commercial software packages have inevitable limitations and disadvantages such as inherent mismatch due to frequency dispersive and time discretization [98]. Therefore, researchers have tried different analytical methods including surface wave modeling [99], Green’s function formulation [100], polarizability [101], and scattering [102]. Such kinds of analytical models have their own advantages over the commercial packages since researcher can customize their calculations according to various designs. Furthermore, some of them can be readily applied to varying plasmonic devices, providing guidance for fabrication and experimental verification. Using simple plasmonic gratings, one can easily achieve the focusing effect. For instance, Verslegers and coworkers fabricated a planar lens formed by slits with different widths etched in a 400 nm thick gold film [103]. Phase delay is introduced by varying gap widths and therefore focusing capability can be realized by elaborately arranging the slits. The curved phase front is realized for the transmitted light wave. As shown in Figure 4a, 3-µm-long slits are defined with 80 nm width in the center to 150 nm on the side symmetrically. Figure 4b,c show measured and calculated results of metafocusing capability of nanoslits superlens which agree well with each other. The experimental result measured by a confocal microscope equipped with a stepper motor (60 nm resolution in the z direction) show focusing distance of 5 µm which is a little bit larger than FDTD simulations. Note that the wavelength is 637 nm for both measurements and simulations. Moreover, active lensing manipulation [104] can be readily achieved by filling the nanoslits with nonlinear media. The focal length can be simply engineered by controlling the intensity of incident light. 

## 3. Focusing via Metasurfaces

In this section, we review recent interesting focusing devices triggered by metasurfaces which have been well studied and the most important advantage of these metasurface-enabled devices over bulky optical components is that they are highly convenient for integration with electronics. Furthermore, these thin metasurface designs can generate geometric phase changes and enable more interesting artificial properties that natural materials do not possess. The basic working mechanism is that gradient metasurfaces can introduce space-variant phase (Pancharatnam–Berry phase) changes and, therefore, they can provide many new features [105,106,107,108,109] and, thus, enable more functional applications in optics and photonics. In addition, they could find wide applications in advanced sensing and diagnostics [110], equivalent-circuit model designs [111], and manipulating visible light [112]. More interesting electromagnetic phenomena such as electric currents and surface waves have also been reported [113,114,115], which have affected the metadevice radiation properties significantly.

### 3.1. 2D Metasurface Lens

**Figure 5:**
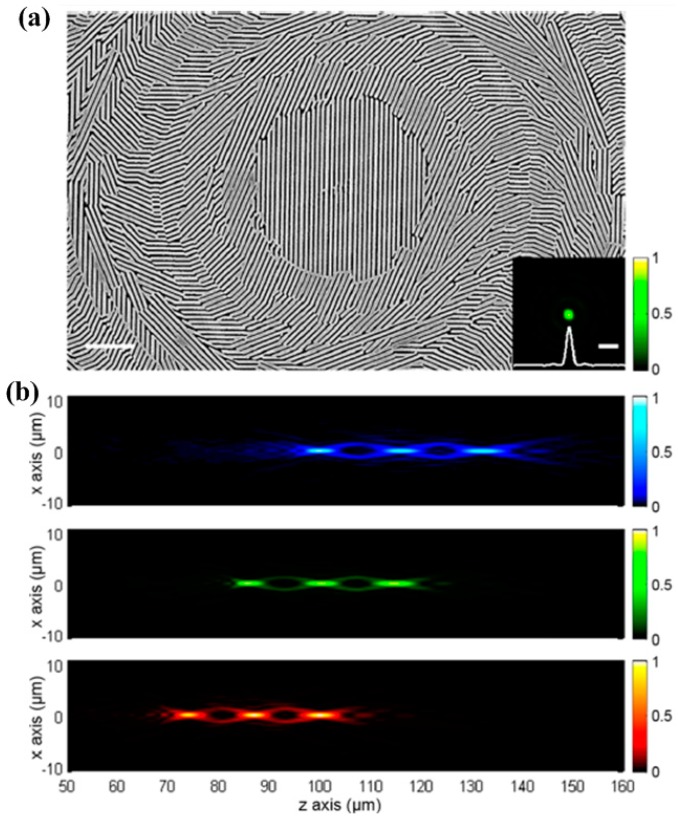
(**a**) SEM topography of the interleaved metasurface containing various sub-elements. (**b**) Measured transmitted intensity profiles for blue, green, and red colors. Reproduced with permission from [123]. Copyright American Chemical Society, 2016.

V-shaped nanoantennae metasurfaces were first reported by Yu and coworkers [116]. After that, various designs have been investigated including fish-bone antennae [117,118,119] and rotating rectangular antennae [120,121,122]. Lin and coworkers demonstrated a multifunctional metasurface lens which can focus light with different wavelengths to a shared focal plane [123]. As shown in Figure 5a, this so-called interleaved metasurface contains various sub-element designs and, therefore, enables multiple functions within a shared aperture. Experimental results (Figure 5b) show that three different wavelengths can focus to a shared focal plane which is around 100 µm behind the metasurface lens. Moreover, it is worth to note that the working principles can be readily applicable to any high-index materials at desired frequencies. 

The measured intensity distributions (Figure 5b) also indicate that the transmitted profiles show different focal spots for various colors (red, green, and blue). This interleaved metasurface is essentially an optical antenna array with different geometrical structures and sub-wavelength spatial spacing [124,125,126]. By designing the phase delay of the radiation field of each nanoantenna, the Huygens principle can be used to achieve arbitrary regulation of the outgoing wavefront. Devices enabled by metasurfaces can achieve precise control of the amplitude, polarization and phase of the incident light field at a scale much smaller than the incident wavelength, which significantly reduces the loss of light propagation in the medium. 

### 3.2. Dynamic Metasurface Lens with Stretchable Substrates

**Figure 6:**
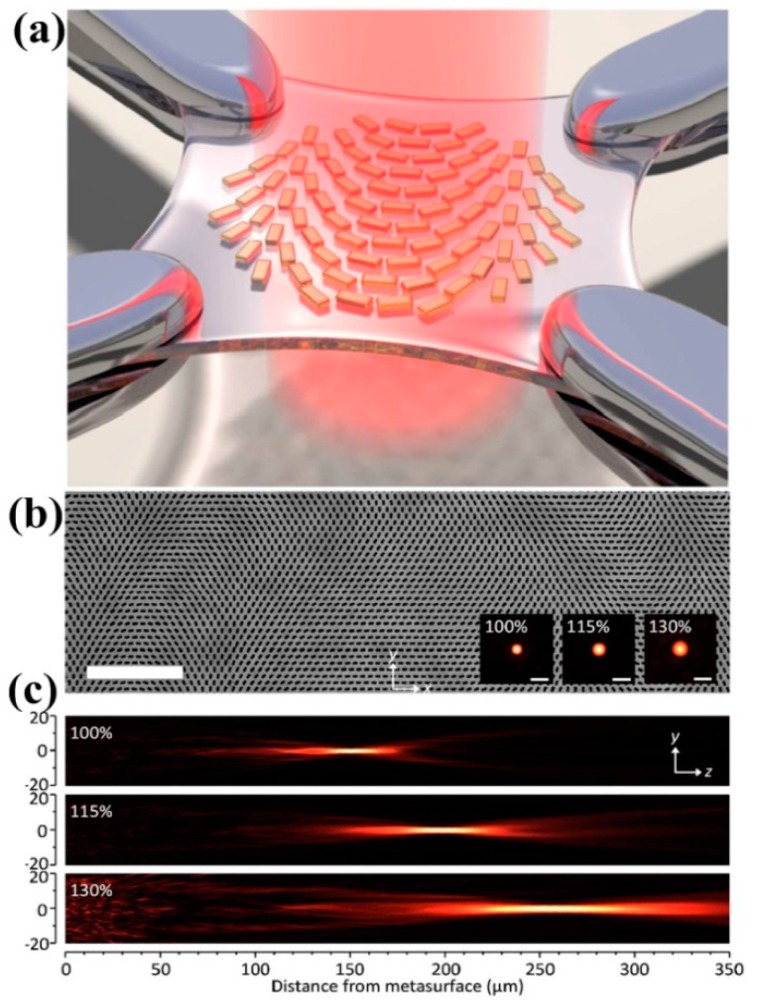
(**a**) Schematic illustration of a metalens array on a stretchable substrate and (**b**) SEM image of the metalens array fabricated by EBL and stripping. (**c**) Measured longitudinal beam profiles generated on the transmission side of the flat zoom lens with different stretch ratios (100%, 115%, and 130%). Note that the metasurface is located at the position of z = 0. Reproduced with permission from [127]. Copyright American Chemical Society, 2016.

Furthermore, a reconfigurable metasurface lens has been experimentally demonstrated with stretchable polydimethylsiloxane (PDMS) substrates [127]. The refraction angle can be manipulated by stretching the substrate mechanically, as shown in Figure 6a. Using electron beam lithography and stripping, a flat lens design is fabricated in PDMS, as shown in Figure 6b. To demonstrate the tunable effect, the longitudinal profiles are reconstructed and the focal length difference can be clearly observed in Figure 6c. Note that such kinds of tunable metasurfaces can regulate the light field with sub-wavelength pixels, which can effectively avoid crosstalk of multi-level diffraction in traditional diffractive optical elements, and they have broad application prospects in micro-integrated photovoltaic systems. In addition, they can perform the same modulation on the light field in a wide band with weak dispersion. These advantages, combined with the superior design flexibility and low-cost manufacturing advantages of metasurfaces, make it a new generation of ultra-thin, ultrasmall-scale light field control devices. These devices can be applied to different wavelengths, leading to a universal component. In short, such kind of metalenses can be readily integrated into many commercially available optical devices.

### 3.3. Near-Field Fish-Bone Metalens

**Figure 7:**
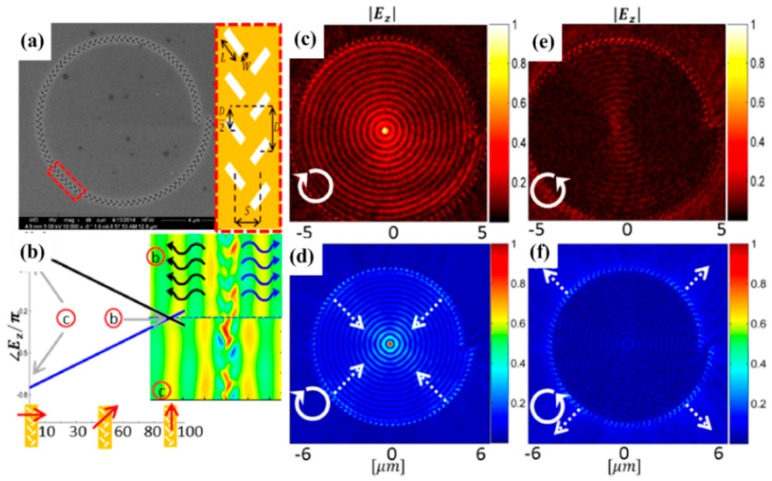
(**a**) SEM and schematic illustration of the fishbone structures. (**b**) Linear phase dependence of surface plasmon polaritons as a function of the polarization orientation. (**c**) Experimental SNOM and (**d**) calculated results for right circularly polarized incidence. (**e**) Experimental SNOM and (**f**) calculated results for left circularly polarized incidence. Reproduced with permission from [128]. Copyright American Chemical Society, 2015.

To further investigate the near field properties of metasurfaces, Spektor and coworkers [128] employed fishbone-shaped nanoantennae and characterized the near-field properties using a scanning near-field optical microscope (SNOM). Fishbone structures were first studied by Lin and coworkers [117] and it was found that the propagation direction could be controlled by the polarization state of the incident light. Different propagation directions can be realized with varying incident beam (circularly polarized light). Using a spiral metasurface design as shown in Figure 7a, one can achieve high efficiency and uniformity focusing function with circularly polarized illumination. Figure 7b plots the phase as a function of the polarization orientation and one can clearly see a linear phase dependence for linear polarizations. Under circularly polarized illumination, however, selective focusing function reveals as shown in Figure 7c (right circularly polarized) and Figure 7e (left circularly polarized), which agree well with FDTD calculations as shown in Figure 7d,f, respectively.

## 4. Potential Practical Applications

### 4.1. Flying Lens for Nanolithography

**Figure 8:**
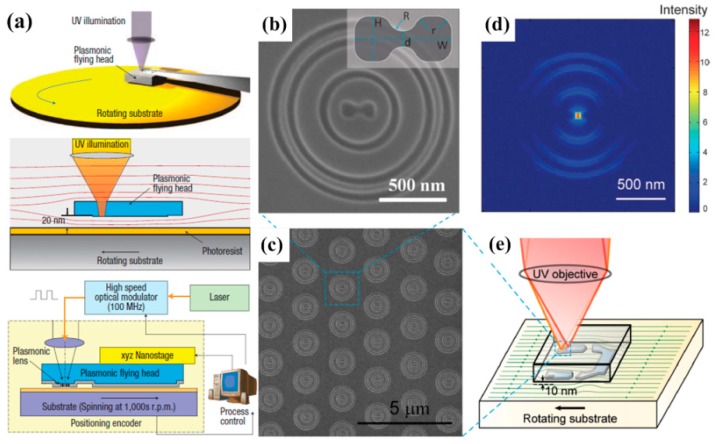
(**a**) Schematic of the maskless nanolithography concept using a flying lens. (**b**) Magnified SEM and (**c**) overview of the plasmonic lens structure. (**d**) Calculated intensity distribution at 10 nm distance away from the lens with 355 nm incident wavelength. (**e**) Schematic of the plasmonic flying head using advanced airbearing surface. Reproduced with permission from [129] and [130]. Copyright Springer Nature, 2008 and 2011.

Figure 8a shows the schematic drawing of a maskless nanolithography system which combines a plasmonic flying head and a rotating substrate [129]. By using an air-bearing slider under ultraviolet illumination, a low-cost and high-throughput nanofabrication technique can be achieved. Laser pulses are manipulated by an optical modulator and the writing position is accurately dominated by a spindle encoder. The spinning substrate can produce a so-called air bearing surface which can provide an aerodynamic lift force, enabling accurate regulation of the nanogap. Further experimental results [130] using a multistage plasmonic lens array, as shown in Figure 8b,c, with a 355 nm wavelength illumination show that 22 nm half pitch resolution can be realized at 7 m/s substrate velocity and 160 MHz laser pulse repetition rate. This kind of low-cost and high-throughput direct writing approach may enable new nanolithography techniques with high speed and high aspect ratio [131]. Moreover, it may be useful for magnetic data storage technology (e.g., heat-assisted magnetic recording) to achieve larger capacities.

### 4.2. Metalens for Fluorescence Imaging

Another important application of a metalens is for biological imaging thanks to their unique capability of wavefront shaping. Most recently, Jang and co-workers experimentally demonstrated a disorder-engineered metalens which can enable the individual input–output responses [132]. Note that the disordered metalens is composed of silicon nitride (SiN_x_) nanoposts which can function as truncated multimode waveguides. Moreover, wavefront shaping using a disorder-engineered metalens can enable access to a wide optical space and stable optical focusing function with high-quality.

**Figure 9:**
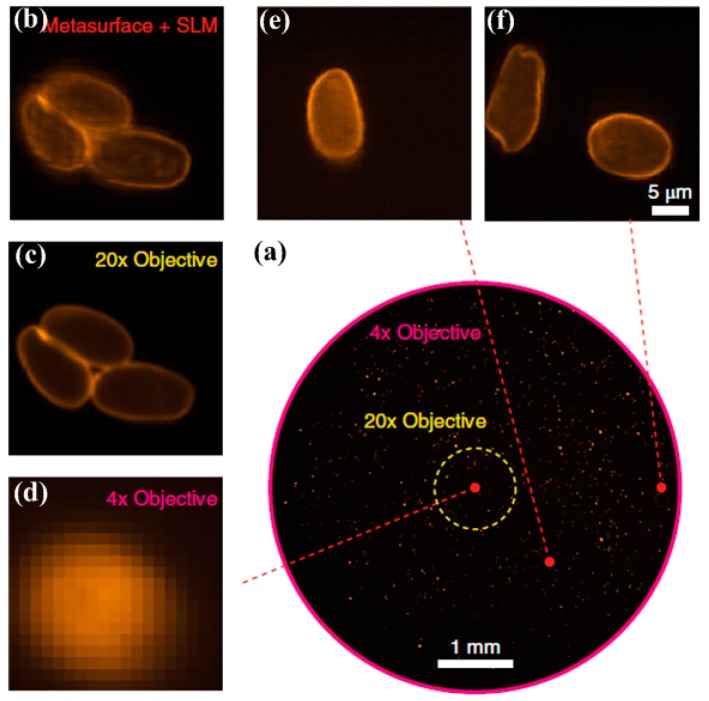
(**a**) Low-resolution image captured by a conventional fluorescence microscope with a 4× objective lens. (**b**) Scanned image obtained with a disordered metalens. (**c**) Ground-truth fluorescence image captured with a 20× objective lens. (**d**) Magnified low-resolution fluorescence image captured with a 4 × objective lens. (**e**, **f**) Images obtained with the disorder-metalens assisted microscope at (x, y) = (1, 1) and (2.5, 0) mm, respectively. Reproduced with permission from [132]. Copyright Springer Nature, 2018.

Figure 9a shows the low-resolution bright-field image of immunofluorescence-labeled parasites using a conventional fluorescence microscope with a 4× objective lens and one can see that the magnified image shown in Figure 9d is blurry. Figure 9b,e,f present the images obtained with a metalens at different locations of (x, y) = (0, 0), (1, 1) and (2.5, 0), respectively. One can see that fine features can be resolved with the capability for high numerical aperture focusing. Figure 9c is the ground-truth fluorescence image captured with a 20× objective lens for comparison. More useful application examples include biomedical sensing [133,134], electromagnetic wave absorbing [135], active beam switching and bifocal zoom lensing [136], telecommunications [137], and so on [138,139]. Most of these metalens-enabled components can be readily used and applied to various optical devices and act as functional instruments which can significantly help improve the device performance. Furthermore, one should note that the new functionalities introduced in this review of these metalensing devices possess high integration compatibility with electronics as well due to their small dimensions, leading to more practical miniaturization designs for optics and nanophotonics.

## 5. Conclusions and Outlook

To conclude, we have reviewed the development of superlensing and metafocusing devices and their potential applications. Due to the recent development of metasurfaces, as well as the exploration of new optical functions, dynamic plasmonic lensing devices and more practical achievements have been developed, paving the way for new plasmonic imaging and focusing technologies. The current developing trends for actively tunable metalens seem to be towards higher modulating speed and large-scale manufacturing or even massive production. However, researchers still need to investigate the feasibility to achieve higher resolution and better optical performance since the main limitations of the metadevices are still challenging, which may need great efforts and investigations, such as developing adaptive metalenses, which can manipulate focal length, astigmatism, and shift simultaneously [140]. In addition, highly tunable devices with low losses are of extreme importance since they may enable more innovations due to their exceptional capability of manipulating electromagnetic waves at the sub-wavelength scale. More future demands and improvements have been proposed including optical modulators, radiative cooling metasurfaces, and new dispersionless flat lenses [114]. Miniaturization of optical metalensing devices will also become an important trend [141] due to their ultrasmall dimensions.
